# Case Report: Use of Tiao Wei Cheng Qi Decoction to relieve partial intestinal obstruction and enable surgical treatment in primary duodenal adenocarcinoma

**DOI:** 10.3389/fonc.2025.1581949

**Published:** 2025-07-15

**Authors:** Ziyao Li, Jiahang Li, Boyang Li, Yingying Sun, Changcheng Wang

**Affiliations:** ^1^ Department of General Surgery, Xiyuan Hospital, China Academy of Chinese Medical Sciences, Beijing, China; ^2^ School of Clinical Medicine, Chengdu University of Traditional Chinese Medicine, Chengdu, Sichuan, China

**Keywords:** primary duodenal adenocarcinoma (PDA), traditional Chinese medicine (TCM), Tiao Wei Cheng Qi Decoction (TWCQD), preoperative treatment, gastrointestinal obstruction

## Abstract

Primary duodenal adenocarcinoma (PDA) is rare and highly invasive malignancies. The onset of the disease is insidious. Patients are often diagnosed when digestive obstruction occurred, leading to poor nutritional status and reduced tolerance for surgical trauma. Additionally, patients commonly experience fluid depletion and gastric drying, which is recognized as a pattern of gastric dryness and fluid injury. This article presents a successful case treated with the modified Decoction for Regulating the Stomach and Resolving Phlegm Formula combined with partial duodenectomy, summarizes both Chinese and Western medical diagnostic and treatment strategies for this disease, and provides valuable clinical experience in treating it.

## Introduction

Primary duodenal adenocarcinoma (PDA) is an exceedingly rare and highly aggressive malignancy, accounting for approximately 0.5% of all gastrointestinal cancers and 45% of small intestinal malignancies (1). Due to its insidious onset and non-specific clinical manifestations, PDA is often diagnosed at advanced stages, leading to poor prognosis and limited treatment options. The disease primarily affects the descending portion of the duodenum, with symptoms such as abdominal pain, vomiting, and digestive obstruction.

.Of particular interest is Tiao Wei Cheng Qi Decoction (TWCQD), a classic prescription from the Shang Han Lun, an ancient Chinese medical text written by the famous physician Zhang Zhongjing. This formula is used to treat constipation and abdominal distension caused by gastrointestinal dryness and heat in the Yangming disorder. Notably, modern medical research has suggested that TWCQD has a significant regulatory effect on gastrointestinal motility (2). Some literature reports that this decoction can effectively improve gastrointestinal dysfunction in patients with acute exacerbation of chronic obstructive pulmonary disease (COPD) (3). However, to date, there have been no reported cases of its use to treat and benefit patients with incomplete obstruction associated with PDA.

This case report presents a 54-year-old male patient with PDA who underwent partial duodenectomy combined with traditional Chinese medicine (TCM) treatment, specifically the modified TWCQD. The integration of surgical intervention and TCM not only alleviated the patient’s symptoms of incomplete obstruction but also improved his nutritional status, facilitating a smoother surgical process and postoperative recovery. This report aims to summarize the diagnostic and therapeutic strategies for PDA, highlighting the potential benefits of combining Western surgical techniques with TCM in managing this rare and complex disease.

## Case presentation

**Figure 1:**
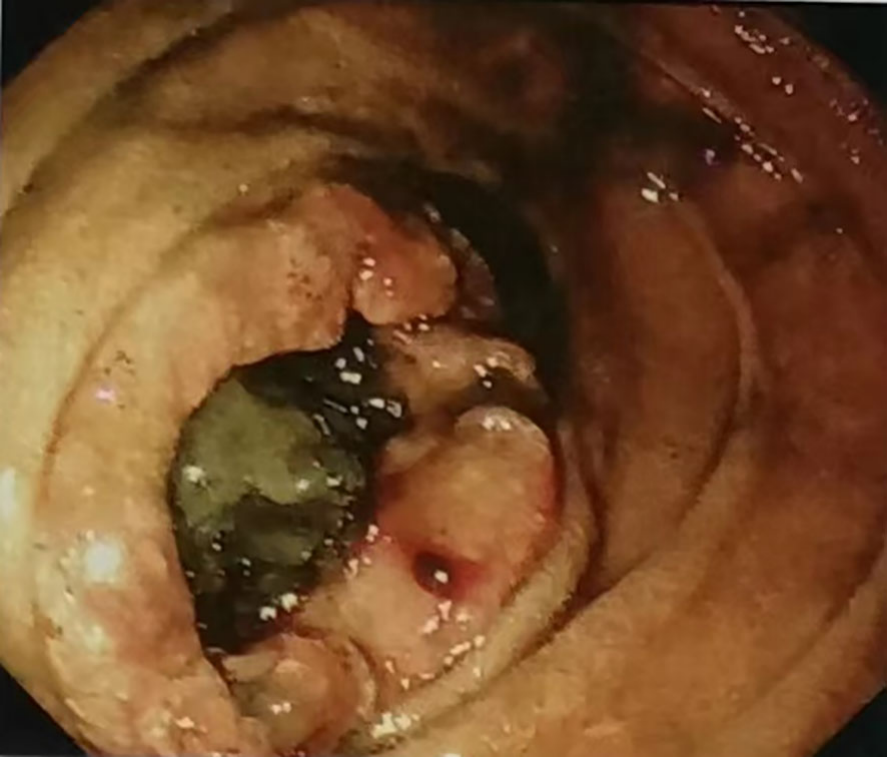
Gastroscopy revealed: a circumferential space-occupying lesion located at the distal end of the duodenal papilla, resulting in marked luminal stenosis and obstruction by food remnants; furthermore, the lesion exhibited an elevated ring-like wall along its periphery.

**Figure 2:**
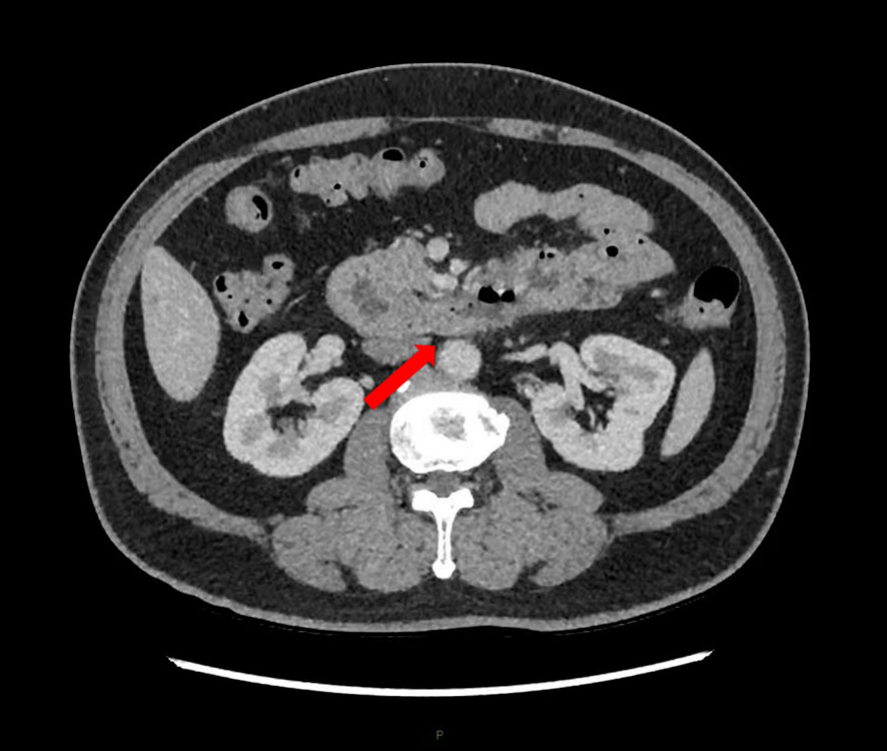
Enhanced abdominal CT revealed thickening and roughness of the horizontal segment wall of duodenum, enlargement and fullness of multiple surrounding lymph nodes, as well as dilatation and effusion in both proximal segments of duodenum and stomach.

A 54-year-old male patient presented with a one-month history of upper abdominal pain and intermittent vomiting. The onset was spontaneous, without any identifiable triggers, and the vomitus consisted of gastric contents. He had received symptomatic treatment for gastroenteritis at a local outpatient clinic but his symptoms remained unresolved. The patient had no significant past medical history, no known family history of cancer or genetic disorders, and no relevant psychosocial or occupational exposures. Half a month ago, the patient presented with worsening vomiting symptoms and significant upper abdominal pain, leading to admission to our hospital’s Department of Internal Medicine for treatment. Gastroscopy revealed (**Figure 1**): a circumferential space-occupying lesion located at the distal end of the duodenal papilla, resulting in marked luminal stenosis and obstruction by food remnants; furthermore, the lesion exhibited an elevated ring-like wall along its periphery. The diagnosis revealed a mass in the descending duodenum, with gastroscopic pathology indicating moderately differentiated adenocarcinoma. The patient was admitted to the surgical department for further treatment due to symptoms of paroxysmal abdominal pain, nausea, inability to eat, exhaustion and absence of defecation. The tongue presents a red hue, the coat appears thin and yellowish, and the pulse is fine. Over the past 3 months, the patient has experienced an approximate weight loss of 15 kg. Hemoglobin levels in blood routine examination were measured at 123 g/L, while tumor markers remained within normal range. Enhanced abdominal CT (**Figure 2**) revealed thickening and roughness of the horizontal segment wall of duodenum, enlargement and fullness of multiple surrounding lymph nodes, as well as dilatation and effusion in both proximal segments of duodenum and stomach. Other imaging examinations revealed no significant abnormalities. The primary diagnosis was a malignant tumor of the duodenum (cT3N2M0) with incomplete obstruction. Following admission, the patient received treatment consisting of gastric tube decompression and total parenteral nutrition, resulting in an improvement in symptoms of abdominal distension and pain. However, the patient was diagnosed with Covid-19 and presented symptoms such as cough and fever. Taking into account the epidemic policy in place at that time and consulting with the anesthesiology department, we made the decision to postpone the operation. The patient presented with incomplete obstruction, and the obstructive symptoms demonstrated significant improvement. Therefore, enteral nutrition was introduced to enhance the nutritional status of the patient. However, after nasogastric feeding, the patient developed significant nausea and vomiting. This was considered to be either due to intestinal edema or intolerance to the enteral nutrition formulation. Based on the patient’s traditional Chinese medicine (TCM) diagnostic assessment, which included the four diagnostic methods (inspection, auscultation and olfaction, inquiry, and palpation), the pattern was identified as Stomach Dryness and Fluid Deficiency. As a result, we prescribed TWCQD with the following formulation: Da Huang (Rhei Radix et Rhizoma) 12g, Zhi Gan Cao (Glycyrrhizae Radix et Rhizoma Praeparatum) 6g, and Mang Xiao (Natrii Sulfas) 9g. The preparation method is as follows: Soak Da Huang and Zhi Gan Cao in 600 mL of cold water for approximately 30 minutes. Then, bring the water to a boil and simmer on low heat for 30 to 45 minutes, or until the liquid reduces to approximately 300 mL. Mang Xiao is added near the end of the decoction process. The decoction was administered via nasogastric tube in two divided doses, with one taken in the morning and the other in the evening. During this period, no other oral intake was provided, except for small amounts of warm water to dissolve and deliver the medication. Within 48 hours of administration, the patient’s symptoms of nausea and abdominal distension were significantly relieved. Subsequently, the nasogastric tube was removed, and TWCQD was continued orally for an additional three days. At the same time, the patient was started on oral enteral nutrition formulas with gradually increasing volume. No adverse reactions such as nausea or vomiting occurred, and the patient’s mental status showed marked improvement. Several days later, the Covid-19 antigen test yielded negative results and respiratory symptoms subsided. Following anesthesia assessment, the patient underwent a resection of the horizontal (D3) ascending portion of the duodenum (D4) under general anesthesia. The tumor was identified at the D3/D4 junction of the duodenum, located approximately 7cm from the greater papilla and with a proximal end about 2cm away and distal end about 10cm away. The duodenum and jejunum, along with the mesentery in the intestinal segment, were excised and anastomosed end-to-end to form a new passage. A jejunal feeding tube was subsequently inserted. Intraoperative pathology confirmed negative resection margins. Postoperative specimen: A 13cm long and 5cm wide segment of small intestinal tissue was obtained, located 2cm from one margin and 9.2cm from the other margin. The specimen exhibited a central depression ulcerated mass measuring 4×4×1.5cm in size with macroscopic invasion of the entire layer, surrounded by raised grey-red dirty tissue with crissum cut surface and impacted fecal stone. Postoperative pathological diagnosis: The duodenal mass was diagnosed as papillary adenocarcinoma, with a tumor size of 4×4×1.5cm and infiltration into the serosa layer. No definite vascular tumor thrombus or nerve invasion was observed, and the tumor budding grade was classified as grade 1. There were no cancer cells found in either surgical margin. Immunohistochemistry results for pT3N0Mx showed positive expression of MLH1, MSH2, MSH6 and PMS2 proteins. Ki67 was expressed in 80% of cells while P53 was wild-type. Her-2 protein was negative and S100 protein was positive. Special staining revealed elastic fiber positivity (x2). The patient resumed normal diet after 7 days and was discharged.

## Discussion

Although the initial staging based on CT was cT3N2M0, postoperative pathology revealed a stage of pT3N0Mx. The discrepancy may be attributed to the overestimation of lymph node involvement by imaging due to inflammation or reactive hyperplasia, which can mimic metastatic disease. This highlights the limitation of radiologic staging in accurately predicting nodal involvement.

For PDA, surgical treatment is currently the most effective approach. Due to the nonspecific early symptoms of this disease, most patients present to hospitals with symptoms of partial or complete upper gastrointestinal obstruction. At this stage, patients often have complications resulting from nutritional deficiencies, such as hypoalbuminemia, which can lead to intestinal edema and worsen gastrointestinal obstruction symptoms. Performing surgery on such patients may increase the difficulty of the operation and the incidence of postoperative complications, such as difficult anastomosis due to intestinal edema during surgery and postoperative anastomotic leaks. Therefore, correcting nutritional deficiencies before surgery would be beneficial for improving the patient’s prognosis. For patients with complete obstruction, preoperative correction can only be achieved through parenteral nutrition. However, for those with only slight narrowing confirmed by preoperative duodenal endoscopy, enteral nutrition can still be used for correction. As seen in the case of the patient in this report, some patients may experience significant discomfort through conventional enteral nutrition, which can prevent the original treatment plan from proceeding as intended. Currently, there are no effective pharmaceutical treatments for this type of situation.

Therefore, we employed traditional TCM to treat the patient by applying syndrome differentiation and treatment principles, specifically using TWCQD. This approach alleviated the patient’s symptoms of incomplete obstruction, enabling the patient to undergo enteral nutrition, improve preoperative malnutrition, and enhance the overall preoperative condition. This provided a foundation for the subsequent radical surgery and rapid postoperative recovery.

The patient in this case presented with a mild Yangming syndrome, with the disease located in the upper gastrointestinal tract, so TWCQDwas selected. This formula, from Zhang Zhongjing’s Shang Han Lun, consists of Da Huang, Gan Cao, and Mang Xiao, mixed in a 4:2:3 ratio (4). Da Huang is a bitter and cold herb that purges heat, but its harsh nature can injure the stomach Qi. By soaking it in wine, its coldness is reduced, and the wine helps it to ascend to reach the affected area and clear the heat from the stomach. Mang Xiao is salty and cold, which helps to clear heat and soften hardness, eliminating dryness and stool from the gastrointestinal tract and restoring the stomach’s downward function. Gan Cao is sweet and gentle, moderating the harsh properties of Da Huang and Mang Xiao, relieving pain, clearing heat, detoxifying, and eliminating turbid Qi from the gastrointestinal tract. As Mr. Ke Qin states, “Xiao Huang moistens the residue in the stomach, allowing Qi to descend; Gan Cao nourishes the stomach’s fluids, allowing Qi to ascend” (5). The three herbs work synergistically, moderating each other’s effects and providing a lasting, gentle therapeutic action. This case demonstrates the formula’s effectiveness, as it harmonizes with the patient’s condition.

Chinese medicine has thousands of years of clinical practice and is a great achievement of the Chinese people, as well as a treasure of ancient Chinese science. The use of multi-drug formulations is a unique approach in clinical Chinese medicine, where multiple drugs work together to exert therapeutic or regulatory pharmacological and toxicological effects. Modern pharmacology has confirmed that TWCQD has detoxifying, heat-clearing, and gastrointestinal-regulating effects (2). This decoction contains several compounds, including six anthraquinones, twelve flavonoids, six glycosides, and seven stilbene glycosides (4). The main active ingredients in this formula include emodin, glycyrrhizin, glycyrrhizic acid, and Mang Xiao. Emodin can promote autophagy, inhibit the expression and activity of pro-apoptotic factors such as Bax, prevent the initiation of cell apoptosis, and protect intestinal barrier function (6). Furthermore, emodin can promote the production of short-chain fatty acids, such as butyrate, acetate, and propionate, by beneficial gut bacteria, thereby enhancing the integrity of the intestinal mucosal barrier, suppressing the release of inflammatory factors, and regulating intestinal immune balance (7). Glycyrrhizin and glycyrrhizic acid, the main active ingredients in licorice, increase the number of intestinal goblet cells, upregulate the expression of tight junction proteins, and reduce intestinal tissue damage and the production of pro-inflammatory factors (8). Mang Xiao, a commonly used purgative, is a natural mineral containing sodium sulfate, first recorded in the Shennong Bencao Jing. Pharmacological studies show that Mang Xiao strongly stimulates the reticuloendothelial system, enhancing its phagocytic ability and anti-inflammatory effects. It increases local blood flow in the intestines through neuro-reflex stimulation, improving local blood circulation, restoring vascular function, rapidly lowering temperature and pain, and alleviating swelling (9). Therefore, from the perspective of modern pharmacology, TWCQD can improve symptoms related to incomplete intestinal obstruction caused by PDA, including anti-inflammatory, anti-swelling, intestinal barrier protection, and maintenance of intestinal function, thereby improving the patient’s overall condition.

Although individual pharmacological actions of compounds such as emodin, glycyrrhizin, and sodium sulfate have been studied, it remains scientifically unclear which components of the TWCQD formulation are primarily responsible for the observed therapeutic effects. The synergistic or antagonistic interactions among these herbal compounds are complex and not yet fully elucidated. Therefore, despite the positive clinical outcome in this case, the underlying mechanisms of action remain speculative. Further pharmacodynamic and mechanistic studies—including component isolation, target validation, and controlled experimental models—are essential to clarify the therapeutic basis of TWCQD and enhance its scientific credibility and international applicability.

We acknowledge that the use of TCM formulations and the principle of syndrome differentiation may be unfamiliar to practitioners outside East Asia. For clinicians interested in integrative approaches, collaboration with trained TCM practitioners is advised. Standardized herbal preparations with traceable sources may also facilitate broader international application and regulatory compliance.

## Conclusion

In conclusion, PDA is extremely rare in clinical practice. The disease has an insidious onset, and patients are often diagnosed when symptoms of tumor-related obstruction appear. These patients may have varying degrees of reduced physical reserves and electrolyte imbalances before surgery. For patients with incomplete obstruction caused by PDA, syndrome differentiation and treatment with Chinese medicine could alleviate preoperative symptoms caused by obstruction, improve the patient’s general condition before surgery, help optimize the timing of surgery, and promote rapid postoperative recovery.

## Data Availability

The original contributions presented in the study are included in the article/supplementary material. Further inquiries can be directed to the corresponding author.
